# Comparison of Global Alignment and Proportion (GAP) Score and SRS-Schwab ASD Classification in the Analysis of Surgical Outcomes for Adult Spinal Deformity

**DOI:** 10.1007/s43465-024-01147-x

**Published:** 2024-04-24

**Authors:** Zhaohan Wang, Bing Wu, Zheng Wang, Kai Song, Yuan Xue, Chuyue Zhang, Yan Wang

**Affiliations:** Chinese PLA General Hospital, No. 28 Fuxing Road, Haidian District, Beijing, 100853 China

**Keywords:** Spinal fusion surgery, Mechanical complications, Revision surgery, Strategy

## Abstract

**Study design:**

The GAP score predicted post-operative mechanical complications more effectively whereas SRS-Schwab classification improved evaluation of postoperative PROMs.

**Objective:**

The study compared the GAP Score and SRS-Schwab Classification in predicting surgical outcomes for adult spinal deformity (ASD) and elucidated whether both systems should be included in the preoperative planning.

**Materials and methods:**

Radiographic measurements and health-related quality of life scores at baseline, 6 weeks after surgery, and the last follow-up were collected from a cohort of 69 ASD patients subjected to long segment spinal fusion surgery after they were grouped by GAP score and SRS-Schwab classification respectively. Fisher's exact test and receiver operator characteristic (ROC) curve analysis was used to compare the incidence of mechanical complications and the discriminant capacity during revision surgery between the two groups. Postoperative patient-reported outcomes measures (PROMs) were compared by one-way ANOVA, and the proportions of MCID achieved for PROMs compared by chi-square test between the two groups.

**Results:**

The overall incidence of mechanical complications and revision surgery were 42% and 8.7%. Both GAP score and its categories predicted mechanical complications and revision surgery, but the GAP score system could not predict the improvements of PROMs. The SRS-Schwab classification could predict the occurrence of postoperative mechanical complications and improvements of postoperative PROMs between the aligned, moderately misaligned and severely misaligned groups (P < 0.05).

**Conclusion:**

Hence, a comprehensive surgical strategy for postoperative planning may improve patients’ quality of life and minimize mechanical complications.

**Graphical Abstract:**

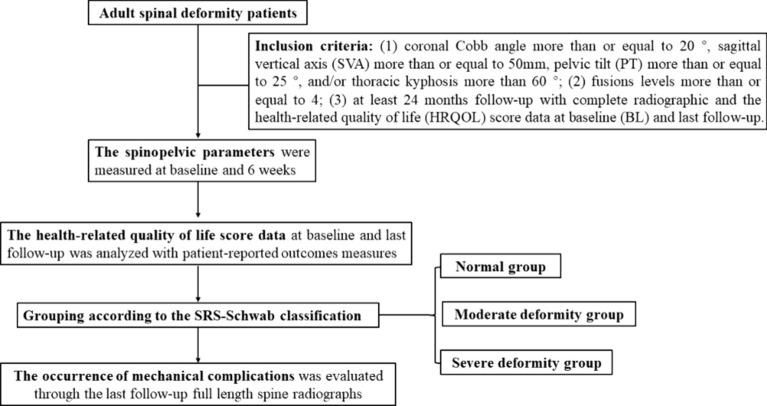

## Introduction

In the current aging population society, the prevalence of adult spinal deformity (ASD) among the elderly over 60 years old is as high as 32–68% [1, 2], which brings an increasingly heavy burden to the public health service system [3]. Current reports suggest that nonoperative treatments offer limited benefits and may not improve the quality of life among symptomatic ASD patients, and reconstructive spine surgery is often recommended [4]. The core concept of reconstructive surgery is to restore the normal sagittal alignment which ensures the least recruitment of compensatory mechanisms and minimal energy expenditure in patients' postoperative daily activities [5]. More importantly, optimal postoperative sagittal alignment can decrease the prevalence of mechanical complications, such as proximal junctional kyphosis/ failure (PJK/PJF), and implant loosening, which can lead to revision surgery and impaired quality of life [6]. To better understand “normal” and “pathologic” alignment and make appropriate correction targets in ASD, various sagittal spine alignment classification frameworks have been proposed. The Scoliosis Research Society (SRS)-Schwab classification and the global alignment and proportion (GAP) score are two representative systems commonly used to guide ASD surgical decisions [7, 8].

The SRS-Schwab classification was based on 3 spinopelvic parameters that were most closely related to health-related quality of life (HRQOL) measures. The 3 modifiers with set thresholds showed the ability to guide decision making between operative and non-operative treatment [9, 10], but the inclusion of this classification for preoperative planning has not yet been proven to prevent mechanical complications. The GAP score is a pelvic-incidence (PI)-based proportional method to analyze the sagittal plane that predicts mechanical complications in ASD patients undergoing reconstructive surgery. This scoring system assists in developing more personalized surgical plans for each ASD patient [8].

The purpose of this study was to compare the GAP Score and SRS-Schwab ASD Classification in predicting surgical outcomes for ASD and clarify whether both systems should be included in preoperative planning.

## Materials and Methods

### Patient Selection

We retrospectively studied a consecutive cohort of ASD patients who underwent long segmental reconstructive fusion surgery by the same surgical team at our hospital (Chinese PLA General Hospital, China) from January 2017 to December 2019. All enrolled patients have signed informed consent forms voluntarily. The study has been approved by the Ethics Review Committee of our hospital with an approval number (S2023-066-01). We collected basic surgical information about the patients, including age, gender, body mass index (BMI), blood loss, operation time, fusion levels, upper and lower instrumented vertebra, osteotomy methods, etc. The inclusion criteria for our study were as follows: (1) coronal Cobb angle more than or equal to 20°, sagittal vertical axis (SVA) more than or equal to 50 mm, pelvic tilt (PT) more than or equal to 25°, and/or thoracic kyphosis more than 60°; (2) fusions levels more than or equal to 4; (3) at least 24 months follow-up with complete radiographic and the health-related quality of life (HRQOL) score data at baseline (BL) and last follow-up.

### Radiographic Parameters and HRQL Score Data Collection

We exported patients’ freestanding, full-length lateral and anteroposterior spine radiographs from electronic files at baseline, 6 weeks postoperatively, and last follow-up. All the radiographs were analyzed with Surgimap (Nemaris Inc, New York, NY) software. We measured spinopelvic parameters at baseline and 6 weeks postoperatively as follows: coronal Cobb angle (CA), pelvic incidence (PI), pelvic tilt (PT), sacral slope (SS), thoracic kyphosis (TK), thoracolumbar kyphosis (TLK), lumbar lordosis (LL), SVA, global tilt (GT), lower lumbar lordosis (LLL). The definition and schematic diagram of the parameters are shown in additional file 1. The measurements were taken by two experienced spine residents independently, and the measurement results with significant differences were determined through discussion among the co-authors.

HRQOL was analyzed with patient-reported outcomes measures (PROMs) at baseline and the last follow-up, including the Oswestry Disability Index (ODI), Short Form-36 (SF-36) outcomes questionnaire, and Scoliosis Research Society-22r questionnaire (SRS-22r). According to studies by Copay and Carreon [11, 12], the minimum clinically significant difference (MCID) of ODI, SF-36, and SRS-22r was determined to appraise the patients’ pre- to postoperative improvements in health-related quality of life. Regarding the MCIDs of each questionnaire, ODI was 12.8 points, SF-36 physical component summary (PCS) was 4.9 points, SRS-22 activity was 0.4 points, and SRS-22 pain was 0.6 points.

### Patient Grouping

The SRS-Schwab classification includes three modifiers: PT, PI minus LL (PI-LL), and SVA. For PT, when its value is less than 20°, between 20°–30°, or more than 30°, it is respectively defined as normal, moderate deformity, or severe deformity; For PI-LL, when its value is less than 10°, between 10°and 20°, or greater than 20°, it is respectively defined as normal, moderate deformity, or severe deformity; For SVA, when its value is less than 4 cm, between 4 cm and 9.5 cm, or greater than 9.5 cm, it is respectively defined as normal, moderate deformity, or severe deformity [7]. For the convenience of grouping and statistical analysis, we used the method reported by Jacobs to assign values to the 3 modifiers [13]. The 3 grades of each modifier: normal, moderate, and severe deformity respectively assigned 1, 2, and 3 points. Then we calculated the total value of the three modifiers. All cases were stratified into the following 3 groups according to the SRS-Schwab classification: the total value of 3 points is defined as normal group, 4–6 points are defined as the moderate deformity group and 7–9 points are defined as the severe deformity group.

The GAP score parameters consist of relative pelvic version (the measured minus the ideal sacral slope, RPV), relative lumbar lordosis (the measured minus the ideal lumbar lordosis, RLL), lordosis distribution index (the L4-S1 lordosis divided by the L1-S1 lordosis multiplied by 100, LDI), relative spinopelvic alignment (the measured minus the ideal global tilt, RSA), and an age factor (AF). The total score of the 5 parameters is the GAP score which divides the patients into 3 groups: a GAP score of 0–2 was defined as the proportioned group; a GAP score of 3–6 was defined as the moderately disproportioned group; those with a GAP score of 7–13 were defined as the severely disproportioned group [8]. Research has found significant age, gender, and racial differences in spinopelvic parameters [14, 15]. Therefore, the ideal spinopelvic parameters we adopted were based on healthy individuals in China reported by Ma et al. [16]. We used different formulas to calculate ideal values according to the patient’s age and gender. For males under 60 years old, the ideal values are SS = 0.63*PI + 7, LL = 0.61*PI + 18, GT = 0.41*PI-9; For women under 60 years old, the ideal values are SS = 0.55*PI + 8, LL = 0.49*PI + 22, GT = 0.45*PI-9; For males aged 60 and above, the ideal values are SS = 0.42*PI + 12, LL = 0.56*PI + 18, GT = 0.39*PI-5; For women over 60 years old, the ideal values are SS = 0.47*PI + 9, LL = 0.29*PI + 28, GT = 0.55*PI-10.

### Mechanical Complications

According to the definition of mechanical complications summarized by Yilgor et al. [8], the occurrence of mechanical complications was evaluated through the last follow-up full length spine radiographs. (1) Proximal junctional kyphosis (PJK) and proximal junctional failure (PJF): proximal junctional angle (PJA) is defined as the angle between the lower endplate of the upper instrumented vertebra (UIV) and the upper endplate of UIV + 2. If PJA is greater or equal to 10° during the last follow-up and has increased more than 10° immediately after surgery, PJK is considered to have occurred; PJF is defined as the occurrence of UIV or UIV + 1 fracture, UIV internal fixation extraction, and/or sagittal subluxation. (2) Distal junctional kyphosis/failure (DJK/DJF) is defined as the angle between the upper endplate of the lower instrumented vertebra (LIV) and the lower endplate of LIV-1 increases by ≥ 10° after surgery, and/or LIV instrumentation is pulled out. (3) Breakage of single or double rods. (4) Other implant-related complications include screw loosening or fracture, displacement of intervertebral cages.

### Statistical Analysis

Statistical analysis was conducted by SPSS (SPSS Inc, Richmond, CA, USA). Continuous variables were presented by mean and standard deviation (SD). For categorical variables, cross-tabulations were generated and Fisher’s exact or Pearson’s Chi-square tests were used to compare distributions. Means of PROMs between different groups were compared by one-way analysis of variance (ANOVA) and Tukey’s method was used to perform post hoc test pairwise. The receiver operator characteristic (ROC) curve analysis was used to compare the discriminant capacity in mechanical complications and revision surgery between the GAP score and SRS-Schwab classification. The statistical analyses were two-sided, and p < 0.05 was considered statistically significant.

## Results

### Demographics and Surgical Description

During the study period, the surgical team in this study completed a total of 77 ASD corrective surgeries, of which 8 cases were excluded due to incomplete postoperative follow-up data. A total of 69 patients were included in the study with an average age of 64.0 ± 8.1 years and an average follow-up time of 44.0 ± 14 months (Table 1). 59 patients (85.5%) were female. All cases underwent posterior-only reconstructive surgery with decompression and transforaminal lumbar interbody fusion (TLIF). The average number of fusion levels was 10.3 ± 2.1, the average surgical duration was 8.0 ± 1.8 h, and the average blood loss was 1014.5 ± 498.2 ml. Among them, 59 cases underwent Smith-Peterson osteotomy (SPO), 8 cases underwent pedicle osteotomy (PSO), and 1 case underwent vertebral column resection (VCR). In this cohort of patients, the overall incidence of mechanical complications was 42% (N = 29), and the incidence of revision surgery was 8.7% (N = 6). The specific description of mechanical complications is shown in Table 1.

**Table 1:** Surgical cohort overview and postoperative mechanical

Complications (N = 69)	
Age (mean ± sd)	64.0 ± 8.1
Female (n, %)	59 (85.5%)
BMI (kg/m^2^)	25.2 ± 3.3
Follow-up time (months)	44.0 ± 14
Surgical description	
Blood loss (ml)	1014.5 ± 498.2
Operation time (h)	8.0 ± 1.8
Fusion levels (n)	10.3 ± 2.1
Method of osteotomy	
Smith-Peterson osteotomy	59
Pedicle subtraction osteotomy	8
Vertebral column resection	2
UIV	
T2	3
T6	9
T9	7
T10	45
L1	5
LIV	
L5	12
S1	2
S2	55
Mechanical complications (N = 29) =)	
Proximal junctional kyphosis (PJK)	7
Proximal junctional failure (PJF)	4
Distal junction failure (DJF)	3
Rod breakage	2
Implant Loosening	13
Revision surgery	6

BMI: Body mass Index; UIV: upper instrumented vertebra; LIV: lower instrumented vertebra

### Baseline and Last Follow-up Radiographic Parameters and Patient-reported Outcomes

Paired samples analysis was performed to compare means of radiographic and patient-reported outcomes at baseline and last follow-up. Compared with baseline (CA:19.5 ± 1.9°, SVA:48.8 ± 65.9 mm, PT:22.9 ± 11.2°, PI-LL:20.2 ± 18.7°, LL:26.2 ± 18.2°), all the spinopelvic parameters were obviously improved at last follow-up (CA:6.2 ± 0.8°, SVA:25.2 ± 39.7 mm, PT:15.0 ± 9.2°, PI-LL:5.5 ± 11.3°, LL:40.5 ± 11.9°, all p < 0.001 except p = 0.002 for SVA) (Table 2). All PROMs improved significantly from baseline to last follow-up, including ODI (46.2 to 28.9), SF-36 PCS score (20.6 to 29.9), SRS-22r total score (2.61 to 3.57), SRS-22r activity score (2.69 to 3.48), SRS-22r pain score (2.35 to 3.37) (all p < 0.001).

**Table 2:** Spinopelvic parameters and health related quality of life scores at baseline and last follow-up

	Base line	Last follow-up	p value
SVA (mm)	48.8 ± 65.9	25.2 ± 39.7	0.002
Coronal Cobb angle°	19.5 ± 1.9	6.2 ± 0.8	< 0.001
PI (°)	46.3 ± 10.7	46.0 ± 10.8	< 0.001
PT (°)	22.9 ± 11.2	15.0 ± 9.2	< 0.001
TK (°)	18.4 ± 13.2	25.5 ± 8.7	< 0.001
TLK (°)	18.7 ± 17.7	9.7 ± 9.2	< 0.001
LL (°)	26.2 ± 18.2	40.5 ± 11.9	< 0.001
PI-LL (°)	20.2 ± 18.7	5.5 ± 11.3	< 0.001
VAS for lumbar	5.6 ± 2.1	3.43 ± 2.2	< 0.001
VAS for lower limb	4.5 ± 2.5	3.2 ± 2.6	< 0.001
ODI	46.2 ± 12.9	28.9 ± 13.1	< 0.001
SF-36 PCS score	20.6 ± 9.2	29.9 ± 10.1	< 0.001
SRS-22r activity score	2.69 ± 0.60	3.48 ± 0.62	< 0.001
SRS-22r pain score	2.35 ± 0.36	3.37 ± 0.64	< 0.001
SRS-22r total score	2.61 ± 0.39	3.57 ± 0.42	< 0.001

### Relationship Between Mechanical Complications and Patient-Reported Outcomes

**Fig. 1:**
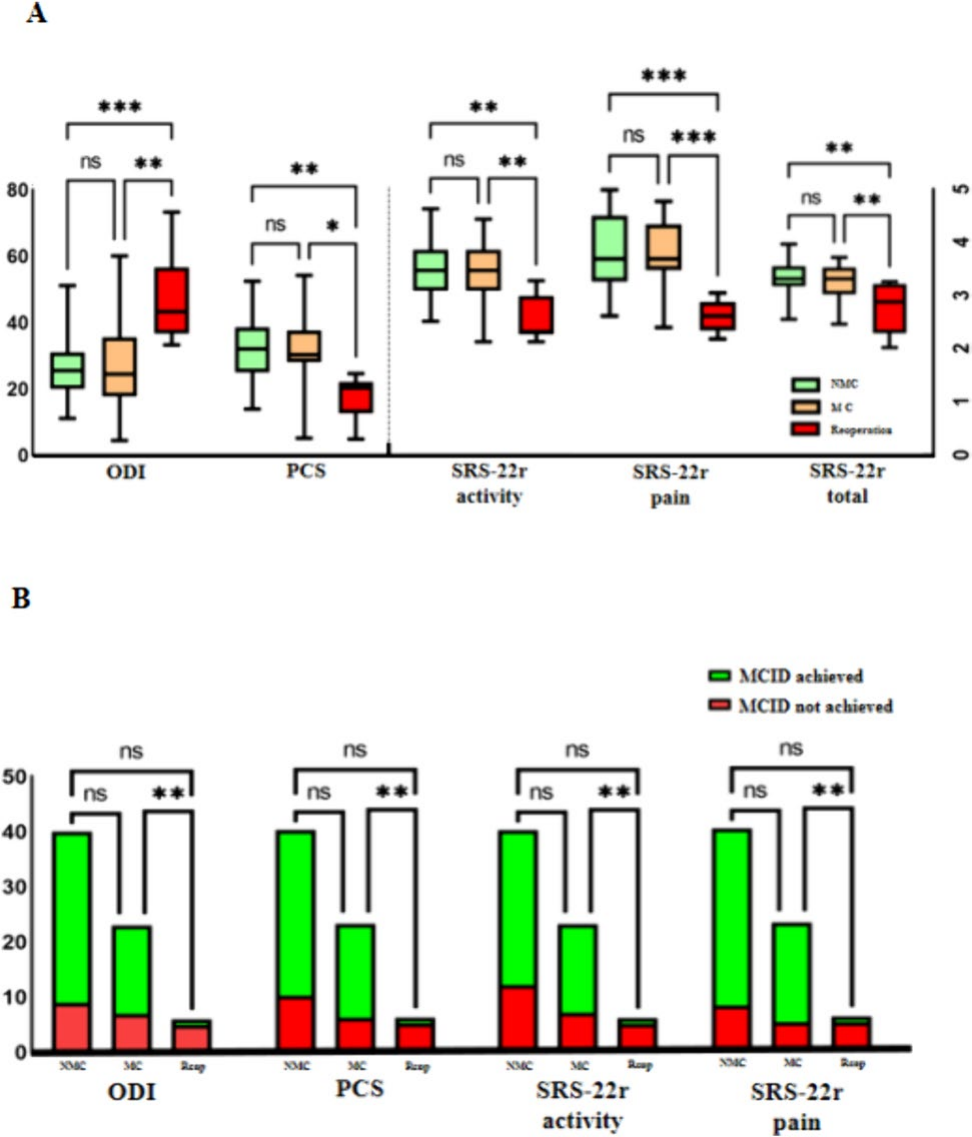
Relationship between mechanical complications and patient-reported outcomes (PROMs). **A** The mean postoperative PROMs per group (mean ± standard deviation, * indicates p < 0.05). **B** The proportions of reaching MCID for each PROMs

Based on mechanical complications and revision surgery, patients were divided into 3 groups: NMC group (patients without mechanical complications), MC group (patients with mechanical complications not re-operated), Reop group (patients receiving revision surgery due to mechanical complications). In the NMC, MC, and Reop groups, there were 40 (58.0%), 23 (33.3%), and 6 (8.7%) patients respectively. By one-way analysis of variance (ANOVA) among the 3 groups, the ODI (NMC:26.6 ± 9.7, MC:28.0 ± 14.8, Reop:47.0 ± 14.2, p = 0.001), SF-36 PCS score (NMC:31.4 ± 9.2, MC:30.6 ± 10.5, Reop:17.5 ± 6.9, p = 0.005) and SRS-22r total score (NMC:3.64 ± 0.35, MC:3.58 ± 0.41, Reop:3.05 ± 0.53, p = 0.004), SRS-22r activity score (NMC:3.57 ± 0.52, MC:3.55 ± 0.67, Reop:2.63 ± 0.46, p = 0.001), SRS-22r pain score (NMC:3.49 ± 0.60, MC:3.41 ± 0.57, Reop:2.40 ± 0.28, p < 0.001) showed statistical differences (Table 3). Using Tukey’s method to perform the post hoc test between the 3 groups, it was found that there was no significant statistical difference in all PROMs between the NMC group and the MC group. However, the Reop group showed significant statistical differences in all PROMs to the NMC group and the MC group (Fig. 1A).

**Table 3:** Relationship between mechanical complications and patient-reported outcomes

	NMC 40	MC 23	Reoperation 6	Statistical value	P value
ODI	26.6 ± 9.7	28.0 ± 14.8	47.0 ± 14.2	F = 7.630	0.001
SF-36 PCS	31.4 ± 9.2	30.6 ± 10.5	17.5 ± 6.9	F = 5.653	0.005
SRS22 activity	3.57 ± 0.52	3.55 ± 0.67	2.63 ± 0.46	F = 7.304	0.001
SRS22 pain	3.49 ± 0.60	3.41 ± 0.57	2.40 ± 0.28	F = 9.506	< 0.001
SRS22 total	3.64 ± 0.35	3.58 ± 0.41	3.05 ± 0.53	F = 6.140	0.004
ODI MCID (n, %)	31 (77.5%)	16 (69.6%)	1 (16.7%)	χ2 = 8.064	0.016
SF-36 PCS MCID (n, %)	30 (75%)	17 (73.90%)	1 (16.7%)	χ2 = 7.576	0.021
SRS-22 activity MCID (n, %)	28 (70%)	16 (69.6%)	1 (16.7%)	χ2 = 6.183	0.046
SRS-22 pain MCID (n, %)	32 (80%)	18 (78.3%)	1 (16.7%)	χ2 = 9.258	0.009

NMC: no mechanical complication group; MC: mechanical complication not reoperated group; Reoperation: mechanical complication with revision surgery group

Each subscript letter denotes a subset of painMCID categories whose column proportions do not differ significantly from each other at the .05 level

Using Fisher's exact test, the proportions of reaching MCID for ODI (NMC:77.5%, MC:69.6%, Reop:16.7%, p = 0.016), SF-36 PCS score (NMC:75.0%, MC:73.9%, Reop:16.7%, p = 0.005), SRS-22r activity score (NMC:70.0%, MC:69.6%, Reop:16.7%, p = 0.021) and SRS-22r pain score (NMC:80.0%, MC:78.3%, Reop:16.7%, p = 0.009) showed statistical differences among the 3 groups (Table 3). Bonferroni-adjusted significance tests were conducted to further compare the proportions of MCID achieved among the 3 groups in pairs. The results showed that there was no significant statistical difference in the proportions of MCID achieved in all PROMs between the NMC group and the MC group. However, the proportions of MCID achieved in all PROMs showed significant statistical differences between the Reop group and the other two groups (Fig. 1B).

### Relationship of Mechanical Complications and Revision Surgery with GAP Score and SRS-Schwab Classification

Based on radiographs at 6 weeks postoperatively, patients were respectively stratified according to GAP score and SRS classification. For the GAP score, the occurrence of mechanical complications between groups was as follows: 4/20 cases in the proportioned group, 20/41 cases in the moderately disproportioned group, and 5/8 cases in the severely disproportioned group. The Chi-square test showed a statistically significant difference in the incidence of mechanical complications between the GAP score groups (χ^2^ = 6.160, p = 0.041). On the contrary, the incidence of mechanical complications between SRS-Schwab Classification groups (aligned group: 27.3%, moderately misalignment group: 45.2%, severely misalignment group: 80%) did not show a statistically significant difference (χ^2^ = 4.860, p = 0.079). In terms of revision surgery, the incidence between different GAP score groups (proportioned group: 0%, moderately disproportioned group: 7.3%, severely disproportioned group:37.5%) presented significant statistical differences (χ^2^ = 7.441, p = 0.013). But among the SRS-Schwab Classification groups, the incidence of revision surgery did not show a statistically significant difference (χ^2^ = 0.901, p = 0.784) (Table 4).

**Table 4:** Relationship of mechanical complications and revision surgery with GAP Score and SRS-Schwab classification

	Mechanical complications		Revision surgery	
	No (40)	Yes (29)	No (63)	Yes (6)
GAP score categories	χ^2^ = 6.160	P = 0.041	χ^2^ = 7.441	P = 0.013
Proportioned	16 (80%)	4 (20%)	20 (100%)	0
Moderately Disproportioned	21 (51.2%)	20 (48.8%)	38 (92.7%)	3 (7.3%)
Severely Disproportioned	3 (37.5%)	5 (62.5%)	5 (62.5%)	3 (37.5%)
SRS-Schwab Categories	χ^2^ = 4.860	P = 0.079	χ^2^ = 0.901	P = 0.784
Aligned	16 (72.7%)	6 (27.3%)	21 (95.5%)	1 (4.5%)
Moderately malalignment	23 (54.8%)	19 (45.2%)	37 (88.1%)	5 (11.9%)
Severely malalignment	1 (20%)	4 (80%)	5 (100%)	0

**Fig. 2:**
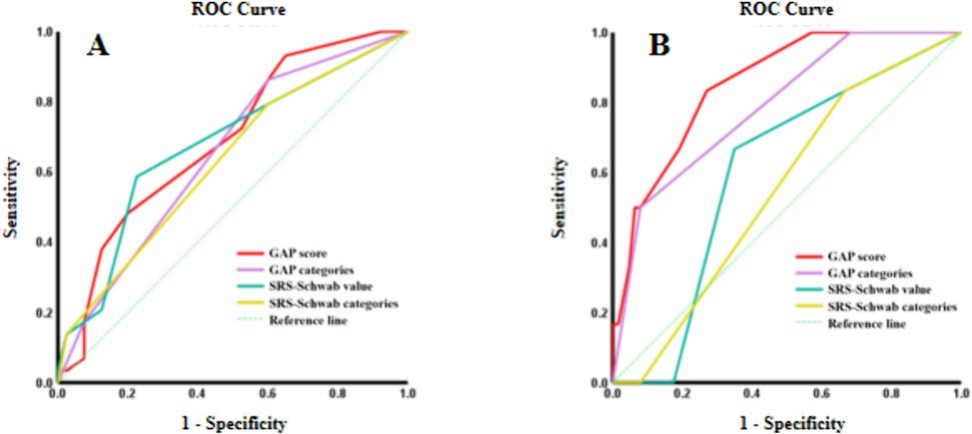
ROC curve of evaluation systems in predicting **A** mechanical complications, **B** revision surgery

The receiver operating characteristic (ROC) curves of the GAP score and its categories, SRS-Schwab classification value, and its categories to predict mechanical complications and revision surgery are shown in Fig. 2. The specific statistical results are shown in Table 5. The ROC curve of GAP score and GAP categories for mechanical complications, with AUC_GAP score_ = 0.691 (P = 0.007) and AUC_GAP categories_ = 0.65 (P = 0.034), indicated that both the GAP score and its categories could effectively predict the occurrence of mechanical complications after ASD reconstructive surgery, and the prediction ability of categories was stronger. The ROC curve of SRS-Schwab classification value and its categories for mechanical complications, with AUC_SRS-Schwab value_ = 0.676 (p = 0.013) and AUC_SRS-Schwab categories_ = 0.628 (p = 0.071), indicated that the value of SRS-Schwab classification had the ability to predict mechanical complications while the categories of SRS-Schwab classification failed to demonstrate predictive ability to MC in statistics (Fig. 2A). In terms of revision surgery, the GAP score and its categories demonstrated moderate accuracy of predictive ability (GAP score: AUC = 0.854, P = 0.004; GAP categories: AUC = 0.79, p = 0.02). In contrast, neither the SRS-Schwab classification value nor its categories showed the predictive ability to revision surgery (SRS-Schwab value: AUC = 0.602, p = 0.412; SRS-Schwab categories AUC = 0.55, p = 0.686) (Fig. 2B).

**Table 5:** Comparison of ROC curve analysis to mechanical complication and revision surgery between GAP Score and SRS-Schwab classification

Test result variable(s)	AUC	Std. Errors	Asymptotic significance	Asymptotic 95% Confidence Interval	
				Lower Bound	Upper Bound
Mechanical complication					
GAP score categories	0.65	0.066	0.034	0.521	0.78
GAP score	0.691	0.064	0.007	0.566	0.815
SRS-Schwab Categories	0.628	0.068	0.071	0.495	0.761
SRS-Schwab value	0.676	0.067	0.013	0.545	0.807
Revision Surgery					
GAP score categories	0.79	0.092	0.02	0.61	0.97
GAP score	0.854	0.068	0.004	0.722	0.987
SRS-Schwab Categories	0.55	0.106	0.686	0.343	0.757
SRS-Schwab value	0.602	0.103	0.412	0.4	0.803

AUC: area under the curve

### Relationship of Postoperative PROMs with GAP Score and SRS-Schwab Classification

Table 6 showed that all the PROMs did not show statistically significant difference among groups divided by GAP Score. Similarly, the proportions of MCID achieved in all PROMs between GAP categories did not show statistically significant differences. On the contrary, in SRS-Schwab Classification groups, all the postoperative PROMs presented a trend of deterioration from the aligned group to the severely misaligned group, and the proportions of MCID achieved in all PROMs showed a decrease in the same group order with statistical significances (all p < 0.05).

**Table 6:** Relationship of postoperative PROMs with GAP Score and SRS-Schwab classification

	GAP score categories					SRS-Schwab categories				
	Proportioned (N = 20)	Moderately disproportioned (N = 41)	Severely disproportioned (N = 8)	Statistical value	P value	Aligned (N = 22)	Moderately malalignment (N = 42)	Severely malalignment (N = 5)	Statistical value	P value
ODI	26.8 ± 10.8	28.0 ± 11.9	38.9 ± 20.5	F = 2.838	P = 0.066	24.4 ± 7.8	28.5 ± 13.1	51.6 ± 16.7	F = 11.410	P < 0.001
SF-36 PCS	30.0 ± 10.1	30.9 ± 9.1	24.5 ± 14.4	F = 1.363	P = 0.263	34.7 ± 7.1	29.0 ± 10.2	16.2 ± 7.5	F = 8.779	P < 0.001
SRS-22r activity	3.65 ± 0.49	3.47 ± 0.61	3.13 ± 0.85	F = 2.143	P = 0.123	3.80 ± 0.37	3.41 ± 0.64	2.64 ± 0.38	F = 9.767	P < 0.001
SRS-22r pain	3.45 ± 0.51	3.42 ± 0.62	2.88 ± 0.87	F = 2.823	P = 0.067	3.57 ± 0.60	3.33 ± 0.65	2.72 ± 0.36	F = 4.106	P = 0.021
SRS-22r total	3.59 ± 0.34	3.62 ± 0.36	3.29 ± 0.71	F = 2.147	P = 0.125	3.71 ± 0.31	3.56 ± 0.43	3.06 ± 0.33	F = 5.682	P = 0.005
ODI MCID (n, %)	15(75%)	29(70.7%)	4(50.0%)	χ2 = 1.773	P = 0.448	18(81.8%)	29(69.0%)	1(20.0%)	χ2 = 6.559	P = 0.030
SF-36 PCS MCID (n, %)	13 (65.0%)	31 (75.6%)	4 (50.0%)	χ2 = 2.459	P = 0.297	20 (90.9%)	27 (64.3%)	1 (20.0%)	χ2 = 10.764	P = 0.005
SRS-22r activity MCID (n, %)	14 (70.0%)	25 (65.9.0%)	4 (50.0%)	χ2 = 1.102	P = 0.658	19 (86.4%)	24 (57.1%)	2 (40.0%)	χ2 = 7.183	P = 0.021
SRS-22r pain MCID (n, %)	13 (65.0%)	34 (82.9%)	4 (50.0%)	χ2 = 4.963	P = 0.077	20 (90.9%)	29 (69%)	2 (40.0%)	χ2 = 6.768	P = 0.031

## Discussion

The results of this study showed that both the GAP score and its categories had the ability to predict the occurrence of mechanical complications and revision surgery, but the GAP score system could not predict the improvements of PROMs. The value of the SRS-Schwab classification could predict the occurrence of postoperative mechanical complications, but it did not have predictive ability for the occurrence of revision surgery. The SRS-Schwab classification could predict the improvements of postoperative PROMs.

Previous studies presented different perspectives on the relationship between mechanical complications and PROMs after ASD surgery. Rothenfloh et al. found that the occurrence of PJK did not affect the clinical efficacy after reconstructive surgery [17]. In the study by Buyuk et al. [18], patients without PJK had a better VAS score and a higher proportion of reaching MCID with ODI compared to patients with PJK, but the difference between the two groups was not statistically significant. Smith et al. believed that postoperative mechanical complications were not the key factor in distinguishing between good and bad clinical outcomes [19]. Although the incidence of postoperative mechanical complications (42%, 29/69) was high in this cohort of patients, only 20.7% (6/29) of the patients with MC underwent revision surgery during an average follow-up of 44.0 ± 14 months. While comparing the PROMs at the last follow-up, it was found that there was no statistically significant difference between the NMC group and the MC group. The above results indicated that the mechanical complications that occurred in this study were mostly mere image findings without influence on surgical outcomes. The ability of the GAP score to predict improvements in postoperative PROMs was not as good as the Schwab classification. The original intention of establishing the GAP score system was to predict the occurrence of mechanical complications after ASD surgery, but many postoperative mechanical complications were mere image findings without clinical significance, which was consistent with our findings. In contrast, the SRS-Schwab classification was found on the basic principle of screening for spinopelvic parameters most closely related to PROMs in ASD patients, which was undoubtedly closely related to patient clinical outcomes.

Our findings showed that both the GAP score and the SRS-Schwab classification had the ability to predict MC which was consistent with the study of Jacobs et al. [13]. The GAP score system was superior to the SRS-Schwab classification system in evaluating postoperative mechanical complications, especially in predicting revision surgery related to MC. In this study, 27.3% (6/22) of the 22 patients who met the SRS-Schwab classification criteria for aligned categories still experienced mechanical complications after surgery. This finding was consistent with Soroceanu’s [20] and Jacobs' [13] research indicating that 31.7% and 38.9% of ASD patients who met the SRS-Schwab criteria still experienced mechanical complications. Many researchers attempting to determine the ideal spine alignment have concluded that the spinopelvic parameters must interact with each other [14, 18]. Therefore, the 3 relatively isolated modifiers with rigid thresholds in the SRS-Schwab classification seemed too simplistic in reflecting the interrelationships between those curvature metrics. An isolated design of each parameter that reaches the so-called normal value without matching other parameters may cause spine stress changes and imbalance, leading to mechanical complications. Pelvic incidence is a morphological parameter that stays the same when other spinopelvic parameters, such as PT, SVA, and LL, vary with spine degenerative changes occur. PI was demonstrated to closely correlate with the other spinopelvic parameters [14] and high PI greater than 50° was proved to relate with PJK [18]. Hence, the PI-based proportional parameters of the GAP score were more in line with the individual variability of human anatomy, which led to harmonious interactions among the spinopelvic parameters.

## Conclusion

Although the incidence of mechanical complications in this study was high, most of them were mere image findings without influence on surgical outcomes. The GAP score had the ability to predict mechanical complications, especially for severe complications that require revision surgery. Although the SRS-Schwab classification was not as effective as the GAP score on the prediction of postoperative mechanical complications, it played a more predictive and guiding role in evaluating improvements in postoperative PROMs. The formulation of surgical strategies for ASD patients’ needs to comprehensively consider the parameters of the GAP score and the SRS-Schwab classification, in order to improve the patient's quality of life while minimizing surgical mechanical complications.

## Data Availability

Not applicable.
